# All-Perovskite Photodetector with Fast Response

**DOI:** 10.1186/s11671-019-3082-z

**Published:** 2019-08-22

**Authors:** Yue Yang, Haitao Dai, Feng Yang, Yating Zhang, Dan Luo, Xiaoli Zhang, Kai Wang, Xiao Wei Sun, Jianquan Yao

**Affiliations:** 1 Tianjin Key Laboratory of Low Dimensional Materials Physics and Preparing Technology, School of Science, Tianjin University, Tianjin, 300072 China; 2 Key Laboratory of Opto-Electronics Information Technology, College of Precision Instruments and Opto-Electronics Engineering, Tianjin University, Tianjin, 300072 China; 3 Department of Electrical & Electronic Engineering, Southern University of Science and Technology, Shenzhen, 518055 China; 4 School of Biomedical Engineering, Southern Medical University, Guangzhou, 510515 China

**Keywords:** Perovskite, CsPbBr_3_ quantum dots, Photodetector, Fast response

## Abstract

Perovskites have attracted substantial attention on account of their excellent physical properties and simple preparation process. Here we demonstrated an improved photodetector based on solution-processing organic-inorganic hybrid perovskite CH_3_NH_3_PbI_3−*x*_Cl_*x*_ layer decorated with CsPbBr_3_ perovskite quantum dots. The CH_3_NH_3_PbI_3−*x*_Cl_*x*_-CsPbBr_3_ photodetector was operated in a visible light region, which appeared high responsivity (*R* = 0.39 A/W), detectivity (D* = 5.43 × 10^9^ Jones), carrier mobility (*μ*_*p*_ = 172 cm^2^ V^−1^ s^−1^ and *μ*_*n*_ = 216 cm^2^ V^−1^ s^−1^), and fast response (rise time 121 μs and fall time 107 μs). The CH_3_NH_3_PbI_3−*x*_Cl_*x*_-CsPbBr_3_ heterostructure is anticipated to find comprehensive applications in future high-performance photoelectronic devices.

**Electronic supplementary material:**

The online version of this article (10.1186/s11671-019-3082-z) contains supplementary material, which is available to authorized users.

## Background

Photodetectors (PDs), which transduce the optical signal into electric information, are one of the key semiconductor devices in many fields, such as optical image sensors, environmental surveillance, electrocommunication and remote sensing technology, etc. [1–4]. Three kinds of devices, i.e., photodiodes, photoconductors, and photo-FETs (field-effect transistors), are commonly adopted to detect optical signals. Especially, photo-FETs are considered as a promising architecture for photodetectors due to their capability to balance high gain and low dark current in comparison with photodiodes and photoconductors.

The photo-FETs have been explored extensively by many groups [5–11]. Generally, to achieve low dark current, a thin active layer is favorable, which renders as depletion layer and can be tuned readily by electric field applied from a gate electrode. However, the thinner the thickness of the active layer is, the lower the optical absorption level is, which leads to low sensitivity. The materials to form an active layer of photo-FETs, therefore, should have high photo-electric conversion efficiency (PECE). Varieties of materials, such as quantum dots (QDs) [12], carbon nanotubes [13], graphene [14], transition metal dichalcogenides (TMDCs) [15], black phosphorus [16], organic molecules, [17] etc., have been employed as active layers for high optical performance of photo-FETs. Until now, halide perovskite has been used widespread as photoactive materials for developing high-performance optoelectronic devices due to its high optical absorption, conversion efficiency, and readily prepared method. Recently, the halide perovskite was also found applications in the high-performance photo-FETs [18–27].

However, even with high PECE material (such as organic/inorganic hybrid perovskite) used as a depletion layer, the light absorption cannot satisfy the practical applications of photo-FETs for efficient gate control. To address the issue, i.e., achieving high gain with low dark current, many solutions have been developed, such as doped with high absorption materials and noble metal nanoparticles for plasmonic enhancement. Among them, the architecture with dye-sensitizer layer prepared on the active layer renders as a promising solution. This architecture can decouple the absorption (in the sensitizer) and charge transport (in the channel) and allows operation of the thin channel layer in full depletion with high optical absorption. Accordingly, a strong absorbing semiconductor is a favorable sensitizer to prepare the high-performance photo-FETs. The QDs, such as PbSe [28], PbS [29], and CdSe [30], have attracted much attention due to the peculiar properties (high quantum yield efficiency, size-sensitive absorption spectrum, etc.) and have been employed in a diversity of high-performance optoelectronic devices.

Very recently, a novel class QDs, i.e., perovskite QDs, have been successfully developed and used in various fields, such as solar cells [31], LEDs [32], and single photon emitters [33]. Considering the requirements of photodetectors, perovskite QDs, i.e., CsPbX_3_(X = Cl, Br, I), is also a suitable sensitizer to enhance the light absorption. As aforementioned, organic-inorganic hybrid perovskite materials have been proved a promising solution for high-performance photo-FETs. In view of the figure of merit of inorganic perovskite quantum dot, we anticipate the all-perovskite device composed of solution-processed CH_3_NH_3_PbI_3−*x*_Cl_*x*_ depletion layer and CsPbBr_3_ QDs sensitizer layer will exhibit excellent performances in responsivity and detectivity. To our knowledge, this composited perovskite photo-FET has not been fully explored before.

In this paper, CH_3_NH_3_PbI_3-x_Cl_x_ perovskite-CsPbBr_3_ QDs hybrid photodetector (CCPD) is prepared with solution-processed strategy. The fabricated photodetector exhibits a wide spectrum span ranging from 400 to 800 nm, high responsivity (0.39 A/W), detectivity (5.43 × 10^9^ Jones), carrier mobility (*μ*_*p*_ = 172 cm^2^ V^−1^ s^−1^ and *μ*_*n*_ = 216 cm^2^ V^−1^ s^−1^), fast response (rise time 121 μs and fall time 107 μs), and good reproducibility. Solution-based CH_3_NH_3_PbI_3−*x*_Cl_*x*_-CsPbBr_3_ heterostructures pave a way for the high-performance optoelectronic device in the UV-visible light region.

## Materials and Methods

### Device Fabrication

First, on the substrate, a commercial silicon wafer (n^+^ Si) with a 300-nm-thick SiO_2_ layer (Suzhou Crystal Silicon Electronic & Technology Co., Ltd), active layer (organic-inorganic hybrid perovskite CH_3_NH_3_PbI_3−*x*_Cl_*x*_) was deposited via spin-coating followed with 90 min post-annealing to resin the film. Subsequently, the sensitized layer, CsPbBr_3_ QDs, was spin-coated layer by layer for three times at 1500 rpm and dried in 60 °anfor 15 min on a hot plate after each spin-coating. The source and drain electrodes were thermally evaporated through a sophisticate shadow mask with a channel length (*L*) of 0.1 mm and a channel width (*W*) of 2.5 mm.

### Materials

*N*,*N*-dimethylformamide (DMF, 99.5%), oleic acid (OA, 90%), 1-octadecene (ODE, 90%), oleylamine (OLA, 90%), PbCl_2_ (99.99%), PbBr_2_ (AR, 99.0%), and CH_3_NH_3_I (98.0%) were purchased from Aladdin.

The details about synthesis of CH_3_NH_3_PbI_3−*x*_Cl_*x*_ perovskite, fabrication of CsPbBr_3_ QDs, and the instrument model are placed at Additional file 1.

## Results and Discussion

**Fig. 1:**
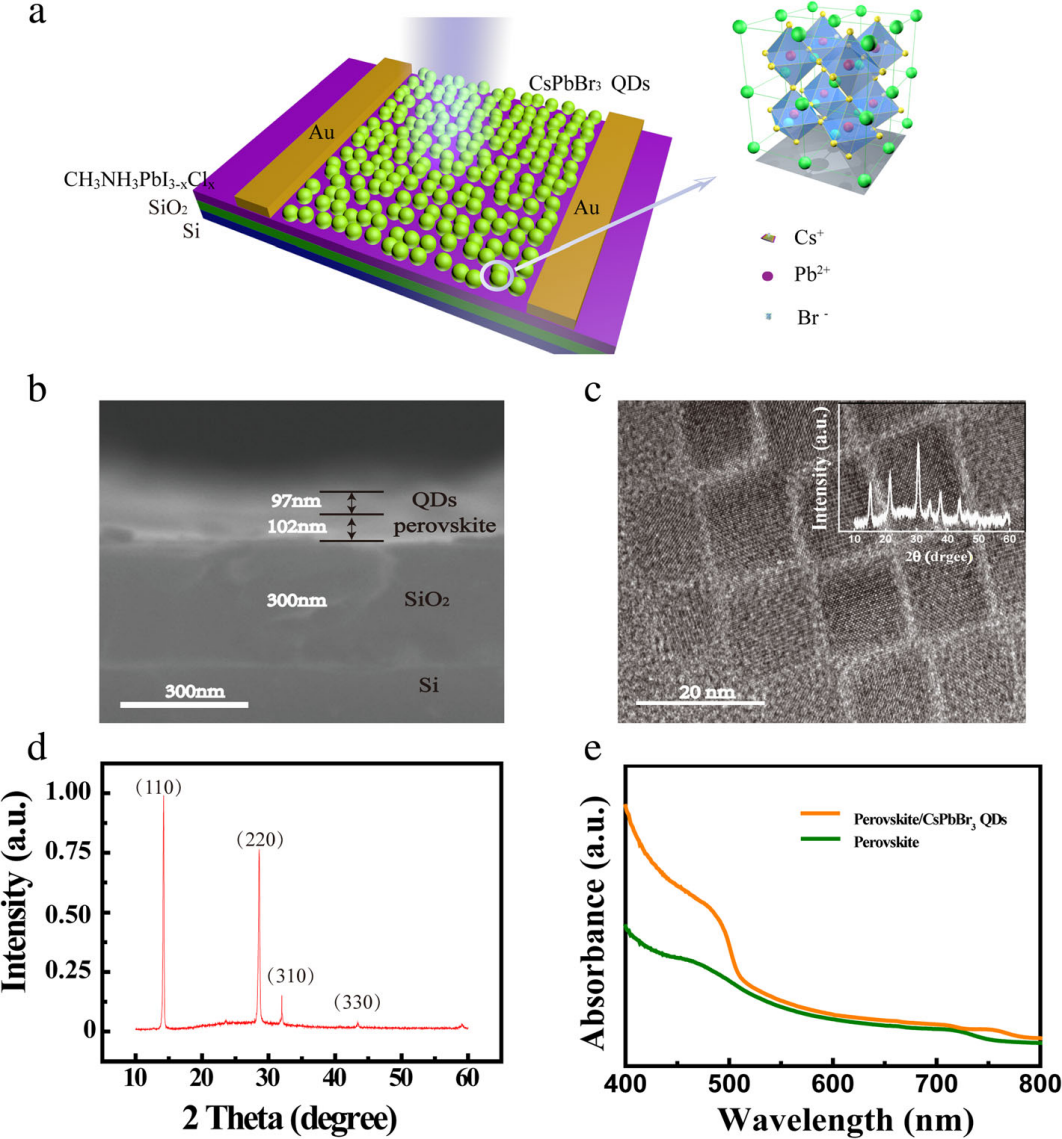
Device structure and related characteristics. **a** Schematic of CCPD. **b** Cross-sectional SEM image of the photodetectors with a scale of 500 nm. **c** TEM image of CsPbBr_3_ QDs with a scale of 20 nm, the inset is XRD spectrum of CsPbBr_3_ QDs. **d** XRD spectrum of CH_3_NH_3_PbI_3−*x*_Cl_*x*_ perovskite film. **e** Optical absorption spectrum of CH_3_NH_3_PbI_3−*x*_Cl_*x*_ perovskite (olive line) and perovskite decorated with CsPbBr_3_ QDs (origin line) on a glass substrate

As shown in Fig. 1a, the photodetectors are composed of a gate electrode, a silicon wafer (n^+^ Si) with a 300-nm-thick SiO_2_ layer (capacitance *C*_*ox*_ of 11.5 nFcm^−2^), active layer (organic-inorganic hybrid perovskite thin film prepared by one step spin-coating solution processing), decorated layer (CsPbBr_3_ QDs), and source and drain electrodes (thermally evaporated through masks). Figure 1 b describes the cross-sectional scanning electron microscopy (SEM) image of the device. The thickness of SiO_2_ dielectric layer is 300 nm, CH_3_NH_3_PbI_3−*x*_Cl_*x*_ perovskite active layer is about 102 nm, and the decorated CsPbBr_3_ QDs layer film is about 97 nm. The diagram shows clearly that the interface between CH_3_NH_3_PbI_3−*x*_Cl_*x*_ perovskite and CsPbBr_3_ QDs is clear and has no intermediate layer, manifesting optimized photoelectric properties. As aforementioned, in photo-FETs, the thickness of semiconducting channel plays a crucial role. Firstly, a thinner active layer is required for tuning the behavior effectively. The thinner perovskite films, however, are prone to produce pinholes, leading to inhomogeneous conduction in the channel. Meanwhile, the thinner active layer means low photon absorption as well. The optimized thickness of the CH_3_NH_3_PbI_3−*x*_Cl_*x*_ film in our device is at about 102 nm. To enhance light-matter interaction in a thinner perovskite device, 97-nm CsPbBr_3_ QD layer, optimum sensitizer with strong absorption is prepared. TEM image of CsPbBr_3_ QDs, in Fig. 1c, shows the uniform particle size and rectangle shape. The inset of Fig. 1c shows the X-ray diffraction (XRD) peaks. The peaks show a typical cubic structure (JCPDS No. 54-0752), which is coincided with the TEM results. Furthermore, to investigate the crystallinity of the CH_3_NH_3_PbI_3−*x*_Cl_*x*_ film, X-ray diffraction (XRD) spectrum of the perovskite film synthesized on a glass substrate chalk up. Figure 1d presents the XRD spectrum, and four characteristic peaks centered at 14.2°, 28.6°, 31.02°, and 43.38° are assigned to the (110), (220), (310), and (330) planes, respectively, indicating that the halide perovskite films possess the expected orthorhombic crystal structure with high crystallinity, which is consistent with the reported literature [34–38].

According to light absorption curves of the CH_3_NH_3_PbI_3−*x*_Cl_*x*_ perovskite (blue line) and perovskite decorated by CsPbBr_3_ QDs (pink line) as shown in Fig. 1e, the decorated CsPbBr_3_ QDs can only enhance the absorption for a narrow range (400–500 nm) in comparison with CH_3_NH_3_PbI_3−*x*_Cl_*x*_ layer solely. Furthermore, we also calculated the bandgap of QDs according to the Tauc equation [39–44] as shown in Additional file 1: Figure S1. The bandgap is about 2.38 eV. The photoluminescence (PL) spectrum of QDs is also shown in Additional file 1: Figure S2, the central wavelength of PL is almost equal to the absorption edge.

**Fig. 2:**
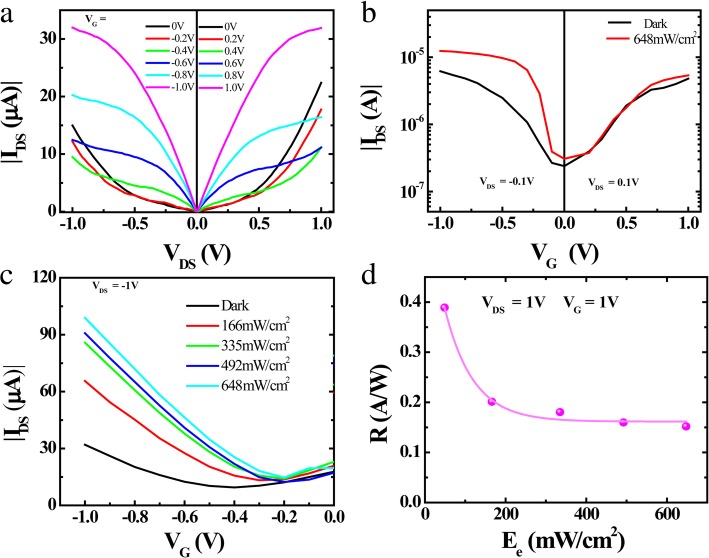
Electrical properties of the perovskite photodetector. **a** Output characteristics at different *V*_*GS*_ in the darkness. **b** Transfer characteristics (*I*_*DS*_ vs *V*_*GS*_) at *V*_*DS*_ = 0.1 V with illumination (red line) and in the darkness (black line). **c** Transfer curve of photodetector as a function of negative gate-source voltage at *V*_*DS*_ = 1 with varying incidence optical powers. **d** Responsivity (*R*) with a relationship of excitation light (*E*_*e*_)

Next, the electrical properties of the devices were explored. Figure 2a describes the *I–V* characteristics of photodetectors with varied gate voltages (0 V, ± 0.2 V, ± 0.4 V, ± 0.6 V, ± 0.8 V, ± 1.0 V) in the dark. There are two states in detectors, according to Fig. 2a. At OFF-state (|*V*_*GS*_| = 0), the spectral lines are linear, and *I*_*DS*_ increases rapidly with the increase of *V*_*DS*_, indicating that a Schottky barrier forms in the device. While at ON-state (|*V*_*GS*_| ≥ 0.4 V), linear-to-saturation current-voltage characteristics appear as the voltage increase, similar to traditional FETs. On account of the excitons remain in the trap states [45] of perovskite which cannot be converted to the photocurrent, leading to the saturation of photocurrent.

The ambipolar performance can be concluded from the transfer characteristics (Fig. 2b) under dark and light illumination, i.e., for both negative *V*_*GS*_ and *V*_*DS*_, the device operates as hole-enhancement mode and, to the contrary, the device operates in the electron-enhancement mode with both positive *V*_*GS*_ and *V*_*DS*_. Owing to the difference of electron potentials, holes separated from photoexcitons generated in heterojunction tend to reside in the perovskite layer. By increasing the incident power density, the transfer rate of holes is higher than electrons. The curve shift towards to positive *V*_*GS*_ in Fig. 2b indicates the heterojunction tend to be *p*-type in this device. Meanwhile, in the linear region, the relationship between field-effect mobility and gate voltage can be extracted with the equation of

1 $$ \mu =\frac{L}{V_{DS}{C}_{ox}W}\frac{\partial {I}_{DS}}{\partial {V}_{GS}} $$

where *L* and *W* are the length and width of the channel, respectively, and *C*_*ox*_ is the capacitance per area. Therefore, the mobility for both holes and electrons can be calculated as 172 cm^2^ V^−1^ s^−1^ and 216 cm^2^ V^−1^ s^−1^. This balanced hole and electron mobility further explains the ambipolar behavior of the device under light illumination.

Figure 2c and d describe the photoelectric properties of the fabricated device. Figure 2c shows the curve of photodetectors as a function of negative gate-source voltage at *V*_*DS*_ = − 1 V with varying incidence optical powers. It is obvious that the device exhibits *n*-type doping behavior. Built-in field at the heterojunction promotes more electron-hole pairs separation and accelerated holes injection into the perovskite channel for negative *V*_*GS*_ and *V*_*DS*_.

Figure 2d exhibits the *R* of the device with the relation of irradiance (*E*_*e*_), in which the wavelength of incident light is 405 nm. As can be seen, the *R* decreases linearly with *E*_*e*_ at irradiant power under 200 mW/cm^2^, while it deviates from linearity at power irradiation greater than 200 mW/cm^2^.

**Fig. 3:**
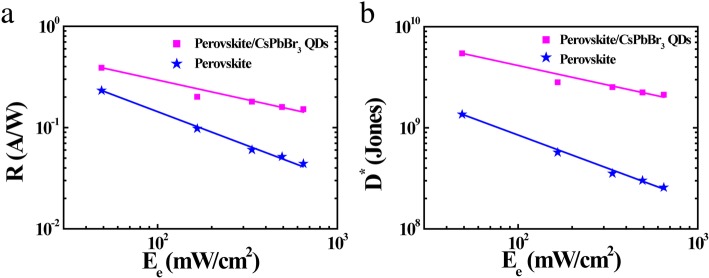
Key parameters of CCPD. **a** *R* of CH_3_NH_3_PbI_3−*x*_Cl_*x*_ perovskite devices (blue line) and perovskite decorated by CsPbBr_3_ QDs devices (pink line). **b** D* as a function of illumination intensity *E*_*e*_. 405-nm continuous laser was used in the test, applied voltage *V*_*DS*_ = *V*_*GS*_ = 1, and irradiance *E*_*e*_ = 0, 166, 335, 492, 648 mW/cm^2^

In order to insight the superior performance of the CCPD. A series of comparisons is necessary. Figure 3a shows the comparison of *R* about the device with the relation of irradiance (*E*_*e*_), in which CH_3_NH_3_PbI_3−*x*_Cl_*x*_ perovskite photodetector (CPD) and CCPD account for the reciprocal function fitting. The *R*, as a figure of merit in photodetector, can be calculated from the formula of

2 $$ R=\frac{I_P}{W\times L\times {E}_e} $$

where *L* is the channel length (0.1 mm), *W* is the channel width (2.5 mm), and *I*_*p*_ is the difference value between light photocurrent and dark photocurrent measured at *V*_*DS*_ = 1 V in the output curve. The maximum *R* is calculated to be 0.39 A/W (in CCPD), obviously larger than that of 0.22 A/W (in CPD). The enhanced responsivity of CCPD is attributed to the CsPbBr_3_ QDs sensitizer with high light absorption and efficient carrier injection in the perovskite layer.

Detectivity (D*) is another key parameter for evaluating the performance of photodetectors. Based on pre-existing responsivity numerical value, the D* versus irradiance (*E*_*e*_) can be estimated by the following equation:

3 $$ {D}^{\ast }=\frac{RA^{\frac{1}{2}}}{{\left(2{eI}_{DS}\right)}^{\frac{1}{2}}} $$

Where *R*, *A*, *e*, and *I*_*DS*_ are the responsivity, available channel area of the devices, charge of an electron, and dark current, respectively. As shown in Fig. 3b, it is clear that D* of CCPD (5.43 × 10^9^ Jones) is notably higher than that of CPD (1.25 × 10^9^ Jones). Further proving sensitized channel material with strongly absorbing CsPbBr_3_ QDs can improve device performance.

Other key parameters representation photodetector’s performance, such as the noise equivalent power (*NEP*), and the gain (*G*) can be given as [46]

4 $$ NEP=\frac{A^{\frac{1}{2}}}{D^{\ast }}\kern0.72em G=\frac{h\nu}{e}R $$

where *R*, *A*, *e*, and *I*_*DS*_ have the same meaning as the previous one. Particularly, when the maximum *R* of CCPD is 0.39 A/W, the D* reached 5.43 × 10^9^ Jones. In this condition, the *NEP* and *G* of this device can be received at an incredibly high value of 9.21 × 10^−12^ W/Hz and 1.197, respectively.

**Fig. 4:**
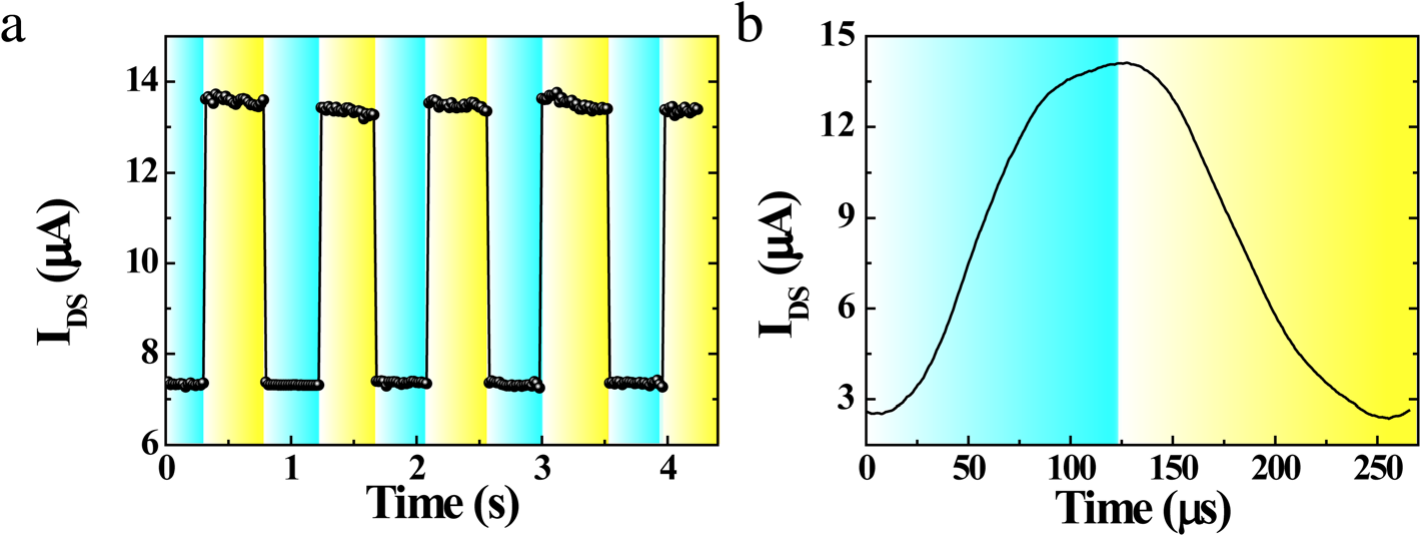
Photoresponse characteristics of CCPD. **a** Current response of the devices under irradiation (*λ* = 405 nm) at *V*_*DS*_ = 1 V and *V*_*GS*_ = 1 V. **b** Temporal photocurrent response of the CCPD under irradiation of 648 mW/cm^2^

Responsibility to optical signals is an important index about efficient carrier transport and collection. Figure 4a shows the drain current with on-off light cycles at a time interval of 20 ms and biased *V*_*DS*_ = 1 V, *V*_*GS*_ = 1 V. As can be seen, the current rises rapidly as soon as the light turned on and reduces quickly while the light turned off, suggesting the good stability and reproducibility in the progress of on-off cycles with light irradiance of 648 mW/cm^2^ at 405 nm. However, a time interval of 20 ms is too long to express the photocurrent response of the device. To calculate the response time of the device, a 4000-Hz pulsed light source is used to irradiate the device. Figure 4b depicts the temporal photocurrent response of the image. The rise and fall times of the photocurrent are ∼121 and ∼107 μs, respectively, indicating an ultrafast response speed than previous reports, as shown in Table 1.

**Table 1:** Comparison of the device performances of CH_3_NH_3_PbI_3−*x*_Cl_*x*_/CsPbBr_3_ device with its single counterparts

Active materials	Photoresponsivity (A W^−1^ )	Rise time (ms)	Fall time (ms)	Ref.
CH_3_NH_3_PbI_3−*x*_Cl_*x*_	620	200	100	[47]
CsPbBr_3_ QDs	0.005	0.20	1.30	[48]
CH_3_NH_3_PbI_3−*x*_Cl_*x*_/CsPbBr_3_	0.39	0.121	0.107	This work

**Fig. 5:**
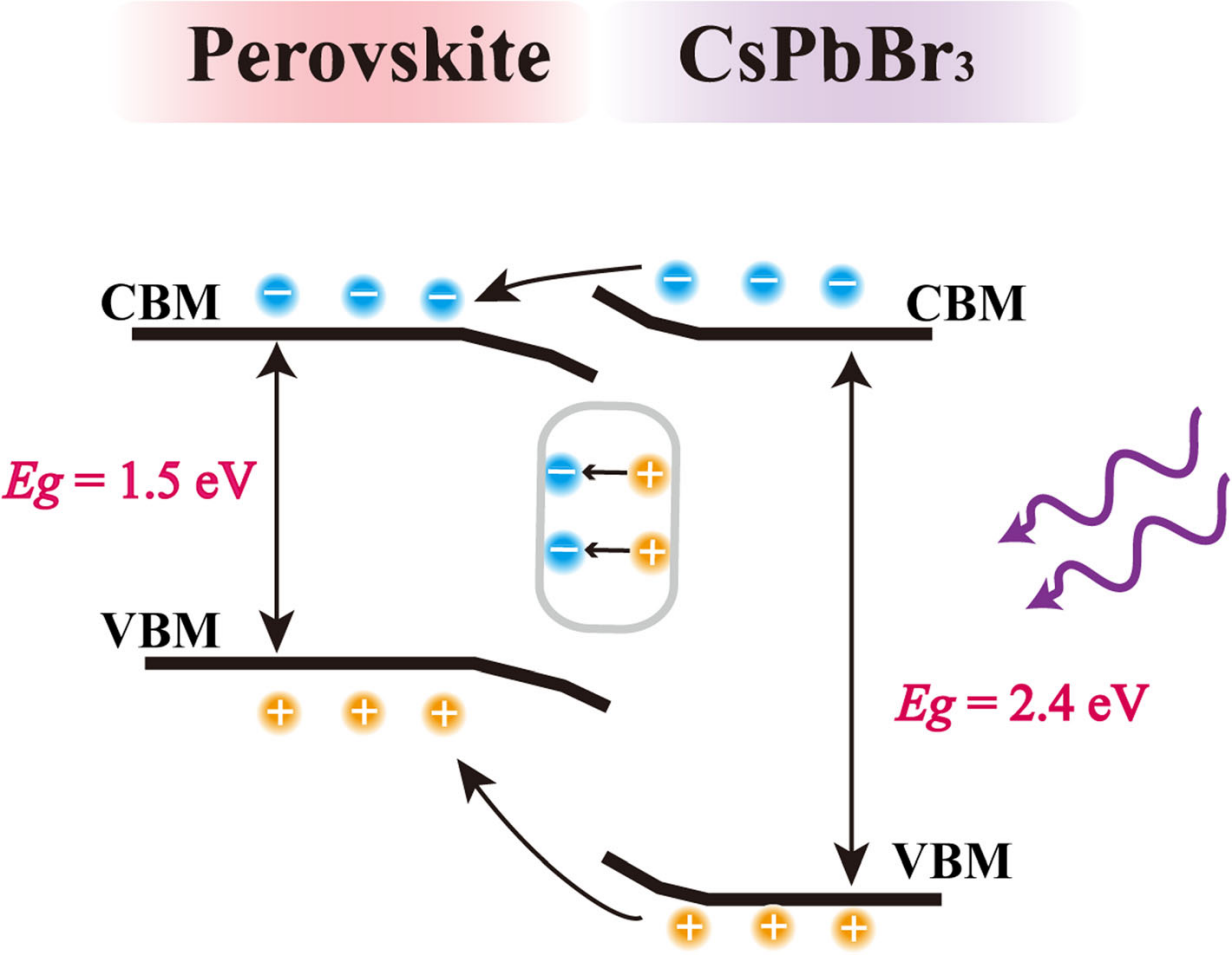
Schematic of the band diagram of hybrid perovskite/CsPbBr_3_ heterostructure

The working principle and interfacial processes of CCPD are schematically showed in Fig. 5. The fabricated detector was excited with 405 nm (3.06 eV) laser, which photon energy is larger than both the hybrid perovskite (1.5 eV) and CsPbBr_3_ (2.4 eV) to ensure the generation of exciton in both layers. As the discrepancy of Fermi energy (*E*_*F*_) of CsPbBr_3_ and hybrid perovskite, the heterojunction would be formed at the interfaces of the two layers, which would mediate or suppress the diffusion of the carriers. Fortunately, the *E*_*F*_ of CsPbBr_3_ is higher than that of hybrid perovskite and leads to an energy configuration as shown in Fig. 5. According to this energy level configuration, the interface can mediate the transport of both carriers from sensitizer layer to active layer, which will enhance the performance of the device. On the other hand, the pristine perovskite has a low density of surface states [49], which lead the band’s easy bending to light absorber layer when the two layers form a heterojunction. This energy level alignment plays an important role in the diffusion of electrons from the sensitizer absorption layer to the perovskite transport layer. The energetic level configuration can accelerate the holes injecting from the CsPbBr_3_ sensitized absorption layer to hybrid perovskite transfer layer, which is coincident with the significant current increases in negative *V*_*GS*_ upon light illumination (shown in Fig. 2b). Meanwhile, the heterojunction in hybrid perovskite/CsPbBr_3_ depletion layer accelerates the separation rate of electron-hole pairs and reduces the separation time, leading to a fast response of the hundred microseconds order.

## Conclusion

In conclusion, we demonstrated highly photosensitive perovskite photodetectors decorated by perovskite QDs. This novel photodetector is operated in a visible light region, which appears high responsivity (*R* = 0.39 A/W), detectivity (D* = 5.43 × 10^9^ Jones), and carrier mobility (*μ*_*p*_ = 172 cm^2^ V^−1^ s^−1^ and *μ*_*n*_ = 216 cm^2^ V^−1^ s^−1^). Meanwhile, the devices also show fast response (rise time 121 μs and fall time 107 μs) and better on-off stability and reproducibility under 405 nm illumination. However, on the one hand, the wide electrode span (hundreds of micrometers) lowers the performances of devices such as photocurrent-related responsivity. Efforts need to be paid to reduce the electrode spacing width for efficient charge transport with less recombination. On the other hand, the short lifetime (few days) of the CCPD remains the severe bottleneck in commercial application. In order to improve the lifetime, further studies will focus on the understanding of ligand effects in the hybrid perovskite-quantum dot devices.

## Additional file

Additional file 1: **Figure S1.** Tauc equation plot. **Figure S2.** PL intensity and absorption curve of CsPbBr_3_ QDs. (DOCX 421 kb)

## Data Availability

The conclusions made in this manuscript are based on the data (main text and figures) presented and shown in this paper.

## Abbreviations

PDs: Photodetectors
CPD: CH_3_NH_3_PbI_3−*x*_Cl_*x*_ perovskite photodetector
CCPD: CH_3_NH_3_PbI_3−*x*_Cl_*x*_-CsPbBr_3_ photodetector
QDs: Quantum dots
FETs: Field-effect transistors
TEM: Transmission electron microscopy
SEM: Cross-sectional scanning electron morphology
XRD: X-ray diffraction
NEP: Noise equivalent power
*G*: Gain
