# Supplementary material for: All-Perovskite Photodetector with Fast Response
Source: Nanoscale Res Lett. 2019 Aug 22;14:291. doi: 10.1186/s11671-019-3082-z (PMC6706520; doi:10.1186/s11671-019-3082-z)
Supplement: Supplementary file 1 — Figure S1. Tauc equation plot. Figure S2. PL intensity and absorption curve of CsPbBr3 QDs. (DOCX 421 kb) [file 11671_2019_3082_MOESM1_ESM.docx]

*1. Synthesis of CH_3_NH_3_PbI_3-x_Cl_x_ perovskite*

The active channel CH_3_NH_3_PbI_3-x_Cl_x_ perovskite thin film was fabricated by the one-step method: PbCl_2_ and CH_3_NH_3_I were dissolved in DMF in a 1:3 molar ratio. Specifically, the concentration of the PbCl_2_ and CH_3_NH_3_I were 0.8 and 2.4 M [32]. The solution was spin-coated onto the 300 nm SiO_2_ layer at 6000 r.p.m. for 60 s to form thin film. Here we adopted multi-step annealing method, that is, annealing the sample at 60℃, 70℃, 85℃ for 10 minutes, respectively. In the end, the sample should be heated up to 110℃ annealing for 1 h on a hot plate to form the high quality film.

*2. Fabrication of CsPbBr_3_ QDs*

The CsPbBr_3_ QDs fabricated [30] with Cs-oleate precursor, which was added Cs_2_CO_3_ (0.0814 g), dried OA (0.25 mL) and dried ODE (4 mL) and into a 25 mL 3-neck flask heated for 1 h at 120℃. Then injected 0.4ml Cs-oleate precursor in another 3-neck flask in 180℃, in which PbBr_3_ (0.069 g) already dissolved in ODE (5 mL) with dried OLA (0.5 mL) and dried OA (0.5 mL). This reaction should take about 10 seconds and then rapidly immersed the flask in a mixture of ice water. Finally, the CsPbBr_3_ QDs can be obtained by centrifugation of the solution in the flask through methylbenzene.

*3. Instrument model*

Transmission electron microscopy (TEM) images were obtained by JEM-100CXII (JEQI, Japan). Cross-sectional scanning electron morphology (SEM) images were taken with HITACHI S-4800. For electric measurements, drain and source electrodes (ground connection) were connected with two output ports of a Keithley 2400 forming bias voltage (*V_DS_*). The drain source current (*I_DS_*) was also measured by a Keithley 2400, and the gate electrode was connected with a constant voltage source HP6030A.

*4. Tauc equation plot*

we calculated the bandgaps of QDs with Tauc equation:

$$\alpha=\frac{A}{h\nu}(h\nu-E_{g})^{n}$$

Where $\alpha$ is the absorption coefficient, A is a constant, $h\nu$ is the photon energy, $E_{g}$ is the band width of semiconductor (band gap), n=1/2 (when the semiconductor is a direct bandgap semiconductor) or n=2 (when the semiconductor is an indirect bandgap semiconductor).


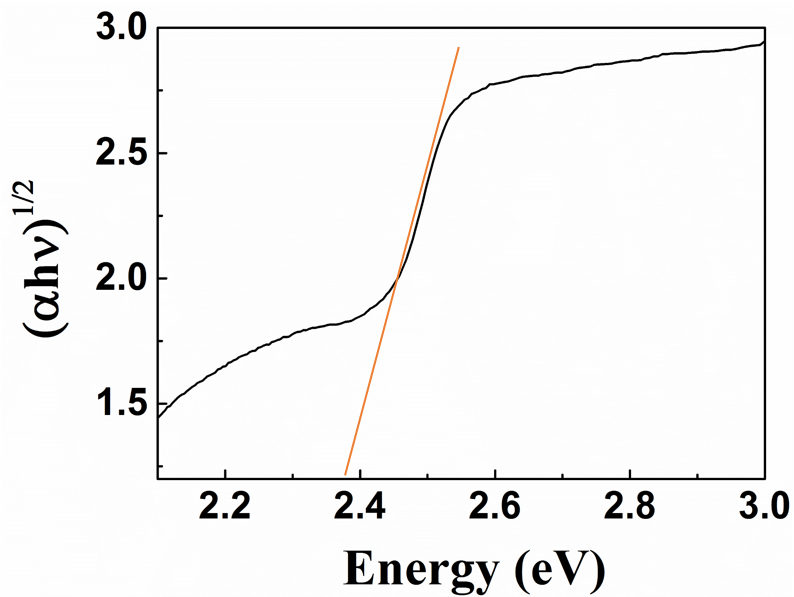


Figure S1. Tauc equation plot.

The intrinsic band gap of the CsPbBr_3_ QDs is estimated to be 2.38 eV.^[1,2]^

*5. PL intensity and absorption curve of CsPbBr_3_ QDs*

Figure S2. PL intensity and absorption curve of CsPbBr_3_ QDs

1. Wang Y, Lv Z, Liao Q et al (2018) Synergies of Electrochemical Metallization and Valance Change in All-Inorganic Perovskite Quantum Dots for Resistive Switching. Adv Mater **30**(28): e1800327.
2. Zhang T, Wang F, Zhang P et al (2019) Low-temperature processed inorganic perovskites for flexible detectors with a broadband photoresponse. Nanoscale **11**(6): 2871-2877
